# Raw Cow Milk Bacterial Consortium as Bioindicator of Circulating Anti-Microbial Resistance (AMR)

**DOI:** 10.3390/ani10122378

**Published:** 2020-12-11

**Authors:** Cristian Piras, Viviana Greco, Enrico Gugliandolo, Alessio Soggiu, Bruno Tilocca, Luigi Bonizzi, Alfonso Zecconi, Rainer Cramer, Domenico Britti, Andrea Urbani, Paola Roncada

**Affiliations:** 1 Department of Health Sciences, “Magna Græcia University” of Catanzaro, Campus Universitario “Salvatore Venuta” Viale Europa, 88100 Catanzaro, Italy; tilocca@unicz.it (B.T.); britti@unicz.it (D.B.); 2 Department of Basic Biotechnological Sciences, Intensivological and Perioperative Clinics, Università Cattolica del Sacro Cuore, Largo Francesco Vito 1, 00168 Roma, Italy; viviana.greco@unicatt.it (V.G.); andrea.urbani@unicatt.it (A.U.); 3 Dipartimento di Scienze di Laboratorio e Infettivologiche, Fondazione Policlinico Universitario Agostino Gemelli IRCCS, Largo A. Gemelli 8, 00168 Roma, Italy; 4 Department of Veterinary Sciences, University of Messina, 98168 Messina, Italy; egugliandolo@unime.it; 5 Surgical and Dental Sciences-One Health Unit, Department of Biomedical, University of Milano, Via Celoria 10, 20133 Milano, Italy; alessio.soggiu@unimi.it (A.S.); luigi.bonizzi@unimi.it (L.B.); alfonso.zecconi@unimi.it (A.Z.); 6 Department of Chemistry, University of Reading, Reading RG6 6DX, UK; r.k.cramer@reading.ac.uk

**Keywords:** proteomics, AMR, raw milk, microbiome, β-lactamase

## Abstract

**Simple Summary:**

Antimicrobials represent useful tools to fight bacterial infections that could harm human and animal health. Antimicrobial resistance occurs naturally or can be induced by the misuse of antibiotics. Its occurrence limits the efficiency of antibiotics and therefore the possibility to treat infections effectively. This can lead to an increasing severity of infectious diseases in humans and animals. Here, we describe the development of a workflow that provides a qualitative representation of the antimicrobial genes that are translated into proteins. Since proteins are ultimately the real effectors, the method herein described demonstrates that those genes are effectively enhancing antimicrobial resistance (AMR). The presented method is independent of any amplification step and provides useful information on the dynamics of the biochemical functions accomplished by the raw milk bacterial consortium.

**Abstract:**

The environment, including animals and animal products, is colonized by bacterial species that are typical and specific of every different ecological niche. Natural and human-related ecological pressure promotes the selection and expression of genes related to antimicrobial resistance (AMR). These genes might be present in a bacterial consortium but might not necessarily be expressed. Their expression could be induced by the presence of antimicrobial compounds that could originate from a given ecological niche or from human activity. In this work, we applied (meta)proteomics analysis of bacterial compartment of raw milk in order to obtain a method that provides a measurement of circulating AMR involved proteins and gathers information about the whole bacterial composition. Results from milk analysis revealed the presence of 29 proteins/proteoforms linked to AMR. The detection of mainly β-lactamases suggests the possibility of using the milk microbiome as a bioindicator for the investigation of AMR. Moreover, it was possible to achieve a culture-free qualitative and functional analysis of raw milk bacterial consortia.

## 1. Introduction

Bacteria are becoming more and more resistant to a greater number of antibiotics. Antimicrobial resistance (AMR) is a trait that can be horizontally transferred by previously resistant bacteria or can be generated by the occurrence of new mutations [1]. The complete panel of AMR genes present within a microbiome is defined as the “resistome”. Moreover, bacteria can be intrinsically resistant to different classes of antibiotics conferring, to a given ecological niche, a certain level of resistance. The bacterial intrinsic resistome is defined as the entirety of elements contributing to antibiotic resistance regardless of previous exposure to antibiotics [2].

For example, soil microorganisms are carriers of resistance genes to many classes of antibiotics independently from human-derived antimicrobial pressure. The intrinsic resistome predates the clinical use of antibiotics posing the question whether AMR occurred earlier than the human antibiotics production and spread [3]. Naturally occurring AMR is related to the biological pressure of every ecological environment/niche that implicates the bacteria-bacteria competition or the bacteria-fungi competition. Penicillin was the first discovered antibiotic and is produced by the fungi of the genus *Penicillium*. Therefore, bacteria-fungi co-existence may have been the driver for the initial production and synthesis of the early forms of beta-lactamases.

Studies based on metagenomics and high-performance genome sequencing broadened the knowledge about bacterial genomes, leading to the discovery of a high concentration of AMR -related genes in many ecological niches. However, the detection of antimicrobial-related genes does not necessarily mean that those genes will be translated into proteins. Antimicrobial genes might be present within a bacterial consortium in the main genome or in the plasmids of the present species, but may remain silent, unless their expression is induced by the presence of antimicrobial compounds in the environment. The genes detected through next generation sequencing (NGS) methods may belong to bacterial species that are dead or unable to replicate. In order to have a deep knowledge of the composition and the biological functions of a microbial consortium, different investigation approaches need to be applied.

The study at protein level (protein expression level) is therefore necessary to assess the full functionality of a given microbial consortium. Mass spectrometry-based proteomics, and specifically metaproteomics, supported by the improved bioinformatic tools, allowed the detection of a high number of different proteins and proteoforms from different organisms in heterogeneous biological samples [4].

Metaproteomics studies represent a challenge for the computational resources because of the large dimension of the databases. Despite this challenge, we have already performed metaproteomics studies which proved to be efficient and reliable for the study of bacterial consortia of hard cheese [5] and of the gut microbiome of newborn mice [6]. The bacterial consortium of hard pasta cheese was enriched using an isoelectric precipitation of caseins to be discarded. The proteomics part was achieved using a bottom-up approach followed by the search against a database including all the bacterial protein sequences obtained from UNIPROT [7]. A similar approach was used to evaluate the diet-related shaping of the whole set of microorganisms present in the gut of newborn mice [6].

In case of raw, unpasteurized milk metaproteome, there are few challenges to overcome to successfully analyze the microbiome. First, unlike metagenomics, it is important to have a robust enrichment step because of the lack of amplification steps for proteins. Second, residual of both milk proteins and somatic cells (which include mainly leukocytes) proteins will be retained in the sample to be analyzed.

For the aforementioned reasons, the challenge of experimentally enriching the raw milk bacterial consortium was addressed with a rapid agitation step and the selective analysis of the bacterial proteins with a bottom-up proteomics approach coupled with database filtering. The main goal was to selectively investigate and demonstrate the expression of proteins related to AMR and, within the same experimental procedure, to evaluate the whole microbial composition up to the genre level.

## 2. Materials and Methods

### 2.1. Milk Sampling

Two bulk tank milk (BTM) samples were collected (each one in duplicate) at distance of 7 days in January 2018 from the official research facility for large animals of University of Milan “Azienda Agraria Didattico-Sperimentale “Angelo Menozzi”—Landriano (Pv)”. The facility counts around 90 lactating cows. These two bulk milk samples were then used for two different extractions named extraction number 1 and extraction number 2.

For this step, 250 mL were taken from the top of the tank using a clean, sanitized dipper after the milk was agitated for 5–10 min as suggested [8]. One aliquot of both samples was delivered refrigerated to ARAL Laboratories for somatic cell count (SCC, 98,000 and 112,000 for the first and the second sample collected) that was performed by certified methods, currently applied by Italian Breeders Association (A.I.A.) on a Fossomatic FC (Foss DK) instrument. The second aliquot collected of each sample was kept at 4 °C and processed within 24 h for bacterial enrichment and metaproteomics analysis.

### 2.2. Bacterial Enrichment for Proteomics Analysis

**Figure 1:**
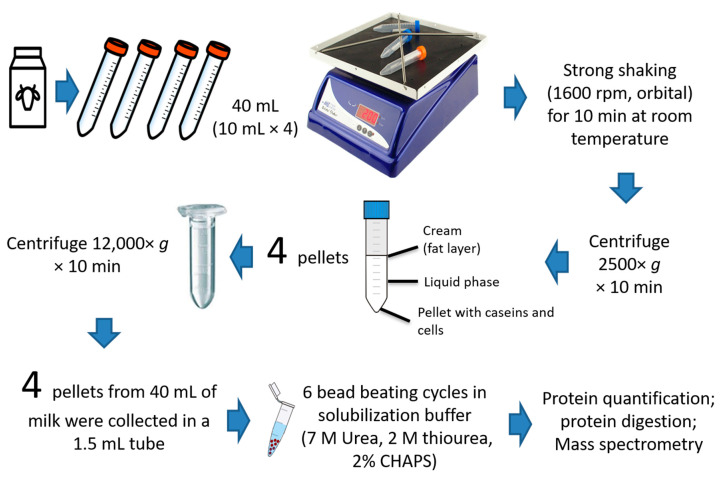
Workflow of the rapid bacterial enrichment method. The experimental phases of the whole procedure prior to the mass spectrometry analysis are described, starting with the separation of the bacterial fraction from the lipid fraction, to the collection and enrichment of the bacterial pellets.

For each sample, 160 mL of fresh milk were divided into sixteen 15 mL tubes (10 mL each tube) and horizontally placed over the plate of a FALC F320 stirrer for 10 min at 1600 rpm (Figure 1).

After this step, the samples were kept in the same vials and centrifuged for 20 min at room temperature at 2500× *g* for cells and bacteria collection. A small red cellular pellet was visible in the bottom of the tube. The top layer (lipids) was removed with a spatula and the supernatant was discarded. Four pellets with the small amount of residual liquid were then gently mixed a pipette and merged in a 2 mL tube. This latter was centrifuged at 12,000× *g* at 4 °C for 20 min. Four of these obtained pellets (coming from 16 original 10 mL tubes) were then collected in one single 2 mL tube and centrifuged again at the same speed. The result is a cellular pellet collected from an original amount of 160 mL of raw milk. This method has been adapted from Brewster and Paul [9]. The supernatant was discarded, and the pellet was then solubilized with 300 µL of solubilization sample buffer (7M UREA, 2M Thiourea, 4% CHAPS). To ensure the complete disruption of the collected bacterial cells the samples were processed with 6 cycles of 1 min bead beating interspersed by a cycle of centrifuge (Figure 1). Bead beating steps were performed by adding to the sample the same amount (1:1 *v*/*w*) of 0.1 mm zyrcounium-sylica beads (300 µg beads added to 300 µL of buffer + the volume of the pellet). The bead beating cycle was performed for 1 min at 4000 rpm in order to avoid overheating. After this step, the samples were centrifuged for 5 min at 12,000× *g* at 4 °C to chill and disperse the foam. This cycle was repeated 6 times. After the 6th cycle, the samples were centrifuged for 20 min and the supernatant was saved in another tube and further processed for proteomics analysis.

### 2.3. Trypsin Digestion and Mass Spectrometry Analysis

Protein Digestion was performed according to the Filter-aided sample preparation (FASP) protocol described by Wiśniewski et al. [10] and optimized by Distler et al. [11] combining both the purification and digestion of the proteins.

Briefly, reduction (DTT 8 mM in urea buffer-8 M urea and 100 mM Tris), alkylation (IAA 50 mM in urea buffer 8 M urea and 100 mM Tris) and digestion by trypsin (final trypsin concentration of 0.01 μg/μL) were performed on filter tubes (Nanosep centrifugal device with Omega membrane-10 K MWCO).

Then, 0.25 µg of each digested samples were loaded in triplicate on a Symmetry C18 5 μm, 180 μm × 20 mm precolumn (Waters Corp., Milford, MA, USA) and subsequently separated by a 120 min reversed phase gradient at 300 nL/min (linear gradient, 2–40% ACN over 90 min) using a HSS T3 C18 1.8 μm, 75 μm × 150 mm nanoscale LC column (Waters Corp.) maintained at 40 °C.

Tryptic peptides were separated on an ACQUITY MClass System (Waters Corp.) and then separated using a High Definition Synapt G2-Si Mass spectrometer (Waters Corp) directly coupled to the chromatographic system.

The protein expression was evaluated by a high definition expression configuration mode (HDMS^E^), a data-independent acquisition (DIA) protocol where ion mobility separation (IMS) was integrated into LC-MS^E^ workflow as described by Marini F. et al. [12].

The mass spectrometer parameters were set as: positive survey polarity of electrospray source (ES+), acquisition mode mass range 50–2000 *m*/*z*, capillary source voltage 3.2 kV, source T 80 °C, cone voltage 40 eV, TOF resolution power 20,000, precursor ion charge state 0.2–4, trap collision energy 4 eV, transfer collision energy 2 eV precursor MS scan time 0.5 s, and fragment MS/MS scan time 1.0 s. All spectra were acquired in IMS cycles with wave height at 40 V, wave velocity of 650 m/s, transfer wave height of 4 V, and transfer wave velocity of 175 m/s.

Data were post-acquisition lock mass corrected using the doubly charged monoisotopic ion of [Glu1]-Fibrinopeptide B (Waters), sampled every 30 s.

### 2.4. Bioinformatics and Metaproteomics

The LC-MS raw data from three replicate experiments for each sample/extraction were processed using the software ProteinLynx Global Server v. 3.0.3 (PLGS, Waters Corp.). The qualitative identification of proteins was obtained by searching two different databases: (i) *bacteria* (UniProt KB/Swiss-Prot Protein Knowledgebase restricted to *all Bacteria taxonomy*) and (ii) The Comprehensive AMR Database (CARD, https://card.mcmaster.ca/) as FASTA files [13,14].

Search parameters were set as: automatic tolerance for precursor ions and for product ions, minimum 1 fragment ions matched per peptide, minimum 3 fragment ions matched per protein, minimum 2 peptide matched per protein, 1 missed cleavage, carbamydomethylation of cysteines and oxidation of methionines as fixed and variable modifications, and a false discovery rate (FDR) of the identification algorithm under 1%.

The protein identifications were based on the detection of more than two fragment ions per peptide, more than two peptides measured per protein.

In addition, in order to validate the proteins of interest obtained by DIA analysis, a targeted label-free strategy was carried out using the freely available Skyline tool (MacCoss Lab Software, https://skyline.ms/project/home/software/Skyline/begin.view).

The qualitative and functional metaproteomics analysis was achieved using the peptides list obtained with PLGS. The obtained list was analyzed with UNIPEPT (https://unipept.ugent.be/) for each different extraction for the qualitative analysis [15].

The Venny 2.1.0 online tool (https://bioinfogp.cnb.csic.es/tools/venny/) was used for comparing lists with Venn Diagrams.

## 3. Results

### 3.1. Cow Milk Microbiome Analysis

As described in the methods section, the first experimental step was necessary to enrich the bacterial fraction. The sample preparation with bacterial enrichment was performed according to the scheme in Figure 1. Raw unpasteurized milk was vigorously agitated to detach the bacterial fraction from the lipids fraction. The samples were subsequently centrifuged to collect the bacterial pellet. This allowed a consistent enrichment of bacteria in a 30-min workflow.

The extraction procedure was performed separately on the first and second sample (extraction 1 and 2, respectively). Each extraction was then analyzed in triplicate via LC-MS/MS DIA integrated with ion mobility separation (IMS).

In order to identify the whole bacterial proteome, the obtained MS datasets were analyzed using different databases: UniProt KB/Swiss-Prot restricted to all reviewed *Bacteria* protein sequences (UniProt KB) and the Comprehensive Antibiotic Resistance Database (CARD) [14].

**Figure 2:**
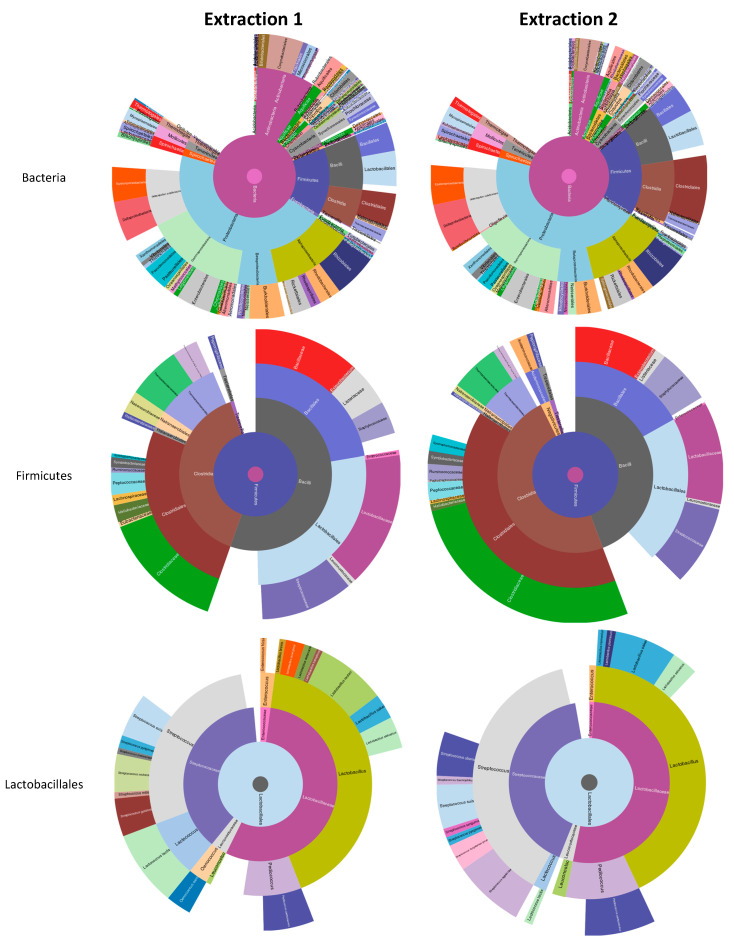
Metaproteomics (Unipept) analysis at the level of the Bacteria domain, Firmicutes phylum, and Lactobacillales order obtained using the peptides identified by searching against the UniProt database restricted to all reviewed bacterial entries.

The technical replicates of the two different extractions were analyzed independently, and the results are shown in Figure 2. The composition of the microbiota showed a low degree of variability between the two extractions. This similarity was consistent up to the genre level (Firmicutes phylum, Lactobacillus genus). However, a higher degree of variability was found when the metaproteomics analysis was undertaken at the species level.

The peptide lists were obtained by searching the raw datasets against the whole bacterial database. The lists were then analyzed to determine the main molecular functions performed by the microbiome just before bacterial lysis. The 10 most probable functions (attributed by Unipept) executed by the whole milk microbiota are listed in Table 1.

### 3.2. Resistome Proteins Analysis

**Figure 3:**
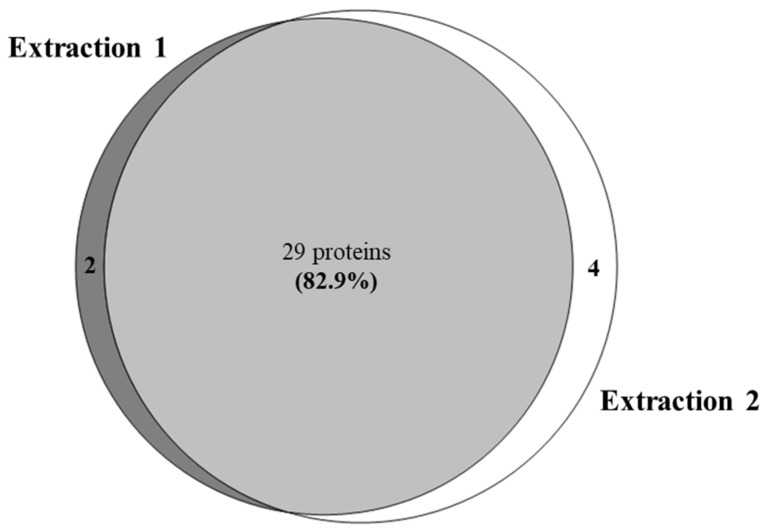
Venn diagram of the proteins identified by searching against the “The Comprehensive Antibiotic Resistance Database” CARD 15.

The same raw MS dataset was then searched against the CARD 15 database. Figure 3 shows the Venn diagram of the proteins identified in the two extractions using the CARD 15 database. Based on the analytical parameters described in the methods, 35 proteins were identified combining both extractions. Specifically, 29 proteins were common to the two extractions corresponding to 82.9% while 5.7% (2 proteins) and 11.4% (4 proteins) were found specifically in extraction 1 and in extraction 2, respectively.

Table 2 shows the proteins commonly detected in both extractions. Those are mainly represented by orthologs of β-lactamases from several bacterial species, e.g., *Klebsiella pneumoniae* and *Escherichia coli*. Among other proteins with AMR potential identified using the CARD15 database there is an isoform of the Aminoglycoside N(6’)-acetyltransferase of *Enterococcus hirae.*

**Figure 4:**
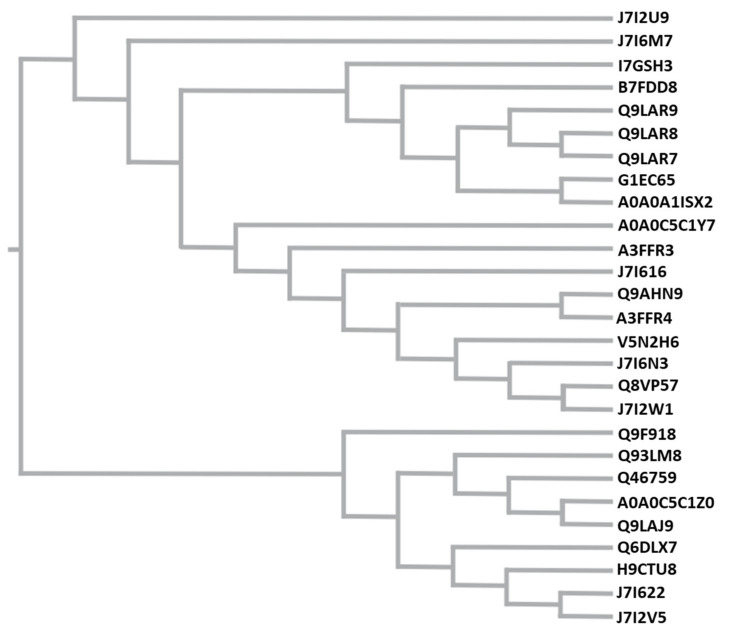
Phylogenetic tree displaying all β-lactamase isoforms detected in the analysed samples using the FastTree function of GenomeNet (https://www.bic.kyoto-u.ac.jp/).

All the β-lactamase isoforms that are present in the analyzed sample are shown in the phylogenetic tree in Figure 4.

In order to validate the DIA results, a targeted label-free strategy was applied to analyze the peptides related to the identified proteins (Table 3).

**Figure 5:**
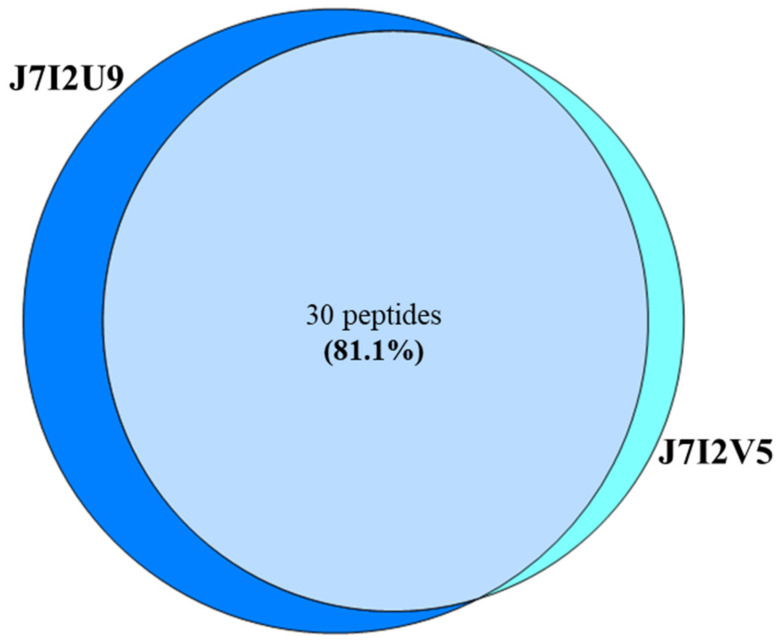
Venn diagram representing the distribution of the tryptic peptides shared between the J7IU9 and J7I2V5 isoforms of β-lactamase.

As shown in Figure 4, it has been possible to differentiate different isoforms of β-lactamase. The most divergent β-lactamase proteoforms showed a 1.4% variability. This produced a difference detectable in 7 tryptic peptides as in Figure 5 and Table 3.

In Table 3, the peptides typical of J7I2U9 and J7I2V5 isoforms are listed with their respective retention times.

## 4. Discussion

Antibiotic resistant bacteria are naturally present in most of the microbial ecosystems. Their prevalence is greater in niches where antibiotics are used as in human beings, farm animals, pets, and closely related environments [16,17,18,19]. Cows are large animals that carry different biological environments rich in many diverse microbiomes. As consequence, they can be carriers of high numbers of antibiotic resistant bacteria and genes. Resistance genes and bacteria commonly do not represent a problem for dairy products because of the hygiene procedures adopted during food processing before their sale (pasteurization, heat treatments, microfiltration, and fermentations during cheese-making) [17]. However, inter- and intra-specific recombination may lead to the creation of single and multi-drug resistant bacteria that might be harmful for the environment and human health [20,21,22]. Once resistance genes are introduced inside an organism, it is difficult to track their flow because of their high rate of genetic recombination [18]. Therefore, it also becomes difficult to link such gene transfers to an eventual antimicrobial resistant infection that occurs in humans or animals.

The mammary gland is probably a sterile environment only before colostrogenesis and milk secretion [23,24]. Once these physiological processes are started and colostrum and milk accumulate into the mammary gland, it becomes an opened environment, and it is colonized by a bacterial microflora [24]. There is a high level of similarity between the milk and the intra mammary microbiome, therefore, the milk microbiome represents a good source of information about the intra-mammary environment. Such environment is altered during mastitis events and produces major changes in the milk microbiome and in the composition/integrity of the milk proteome [25,26].

The most frequently used approach for the study of the milk microbiome is 16 s rRNA sequencing which has also been applied to the study of mastitis [27,28,29]. In the past, it was widely accepted that mastitis is caused by one or a maximum of two bacterial pathogens. However, with the contribution of NGS technologies, it was possible to demonstrate that dysbiosis can be considered as a causative factor for both intra-mammary infections and mastitis itself [24].

Although NGS methods are highly sensitive and accurate in providing information about the composition of the microbiota, they mostly fail to provide information about the functionality of expressed genes. With this technology it is not possible to understand whether these genes are expressed up to the protein level or not. On the contrary, metaproteomic approaches detect protein expression and function.

Efficient bacterial enrichment represents the first step for a successful metaproteomic analysis. For this reason, we adopted a method to enrich the bacterial fraction according to Brewster and Paul [9]. Bacterial binding to the cream layer can be counterproductive for the analysis of the whole bacterial consortium because a relevant part of the microbiome could partition into the cream layer. The agitation step introduced at the beginning of the workflow allowed the collection of the bacterial pellet with a simple centrifugation step. As demonstrated by the aforementioned authors [9], this step facilitates the bacterial detachment from the cream layer resulting in 95% recovery of the viable form. The remaining 5% may still partition with the cream layer or loose viability. As specified in the Methods section, three subsequent centrifugation steps allowed the collection of the bacterial fraction of 160 mL of raw, non-pasteurized, and non-homogenized milk.

Bead beating for bacterial lysis, the FASP method [10,30] for the purification of the protein digestion and DIA IMS mass spectrometry analysis allowed enough dataset depth to study the composition of the bacterial consortia. This analysis was possible up to the genus level providing a qualitative picture of the raw milk’s microbiome (Figure 2). The two different extractions yielded overlapping results highlighting proteobacteria and firmicutes as the two main fila present and bacilli and clostridia as the two dominant classes of firmicutes phylum. The genre lactobacillus, together with streptococcus, were the main genres with aerobic metabolism of firmicutes phylum. ATP binding, DNA binding, and metal ion binding were the three main represented functions that emerged as dominant in this analysis.

On the side of AMR, as can be seen in Table 1, computational analysis carried out in the experiment allowed the detection of at least two proteins involved in AMR. One of them is the β-lactamase that belongs from *Klebsiella pneumoniae*, *Escherichia coli,* or *Acinetobacter baumannii*. β-lactamase producing bacteria can be found in the environment as water sources [31], wastewater [32], and drinking water [33]. This poses the concern about the presence of AMR genes as environmental pollutants that could easily enter the animal and human feeding chain [34]. In this case, the detection of β-lactamase produced by the milk microbiome provides proof that these resistance genes are present among the libraries of this bacterial consortium. Surprisingly, these genes are being translated and expressed to protein level at a considerable amount that can be found with our culture-and induction-free proteomics experiments. This supports the hypothesis that a constant level of β-lactamic metabolizing activity might be often present in complex microbiomes. As Figure 4 and Figure 5 demonstrate, β-lactamic activity was not only due to one isoform of β-lactamase, but to several isoforms. The most divergent isoforms of β-lactamase have 98.6% homology and are different by 4 amminoacids, including one arginine and one lysine substitution, which contribute to the different tryptic digestion profile.

Lactamase activity was not the only resistance mechanism that was detected. Even if in minor amounts, Aminoglycoside N(6′)-acetyltransferase presence was found in the metaproteome of milk. This protein catalyzes the acetylation of aminoglycosides conferring resistance to antibiotics containing the purpurosamine ring including amikacin, kanamycin and tobramycin [35].

## 5. Conclusions

The presented results demonstrated the presence of proteins clearly involved in bacterial resistance. All experiments were performed without any antibiotic induction except for the ones that might be already present in the given ecological niche. The separation of bacterial proteins using a modified precipitation and extraction method combined with bottom-up proteomics allowed the detection of different β-lactamase isoforms. The simultaneous metaproteomics study provided useful information about the taxonomy and the physiological functions of the microbiota.

This method could be easily applied to the study of AMR pattern, bacterial composition and functionality of complex microbiomes. In the field of animal production, it could present an important analytical tool for the study of bulk milk. This study is limited by its application to the characterization of the metaproteome and the resistome of bulk milk of the research facility of University of Milan. Thus, even if the environment is well controlled, does not take into account the possible temporal and geographical variability.

## Figures and Tables

**Table 1:** The 10 most represented Gene Ontology (GO) molecular functions of the analyzed microbiome.

Peptides	GO Term	Name
4001	GO:0005524	ATP binding
1985	GO:0003677	DNA binding
1595	GO:0046872	metal ion binding
1164	GO:0000287	magnesium ion binding
1061	GO:0008270	zinc ion binding
620	GO:0003899	DNA-directed 5′-3′ RNA polymerase activity
514	GO:0016787	hydrolase activity
511	GO:0000049	tRNA binding
481	GO:0005525	GTP binding
473	GO:0046933	proton-transporting ATP synthase activity, rotational mechanism

**Table 2:** List of the different β-lactamase isoforms detected using the CARD 15 resistome database.

29 Common Elements in “Extraction 1” and “Extraction 2”:			
Protein.Entry	Protein.Accession	Protein.Description	Uniprot
ARO:3001066	AAA87176.1	SHV-7 [*Escherichia coli*]	Q46759
ARO:3001077	AAF34333.1	SHV-19 [*Klebsiella pneumoniae*]	Q9LAR9
ARO:3001078	AAF34334.1	SHV-20 [*Klebsiella pneumoniae*]	Q9LAR8
ARO:3001079	AAF34335.1	SHV-21 [*Klebsiella pneumoniae*]	Q9LAR7
ARO:3001076	AAF64386.1	SHV-18 [*Klebsiella pneumoniae*]	Q9LAJ9
ARO:3001073	AAG17550.1	SHV-14 [*Klebsiella pneumoniae*]	Q9F918
ARO:3001087	AAG49894.1	SHV-29 [*Klebsiella pneumoniae*]	Q9AHN9
ARO:3001092	AAK64187.1	SHV-34 [*Escherichia coli*]	Q93LM8
ARO:3001093	AAL68926.1	SHV-35 [*Klebsiella pneumoniae*]	Q8VP57
ARO:3001088	AAT75225.1	SHV-30 [*Enterobacter cloacae*]	Q6DLX7
ARO:3001146	ABN49111.1	SHV-94 [*Klebsiella pneumoniae*]	A3FFR3
ARO:3001148	ABN49112.1	SHV-96 [*Acinetobacter baumannii*]	A3FFR4
ARO:3001182	AEK80394.1	SHV-140 [*Klebsiella pneumoniae*]	G1EC65
ARO:3001183	AFC60795.1	SHV-141 [*Klebsiella pneumoniae*]	H9CTU8
ARO:3001188	AFQ23955.1	SHV-149 [*Klebsiella pneumoniae*]	J7I2U9
ARO:3001190	AFQ23957.1	SHV-151 [*Klebsiella pneumoniae*]	J7I6M7
ARO:3001193	AFQ23960.1	SHV-154 [*Klebsiella pneumoniae*]	J7I2V5
ARO:3001195	AFQ23962.1	SHV-156 [*Klebsiella pneumoniae*]	J7I6N3
ARO:3001197	AFQ23964.1	SHV-158 [*Klebsiella pneumoniae*]	J7I616
ARO:3001198	AFQ23965.1	SHV-159 [*Klebsiella pneumoniae*]	J7I2W1
ARO:3001200	AFQ23967.1	SHV-161 [*Klebsiella pneumoniae*]	J7I616
ARO:3001202	AFQ23969.1	SHV-163 [*Klebsiella pneumoniae*]	J7I622
ARO:3001357	AHA80959.1	SHV-173 [*Klebsiella pneumoniae*]	V5N2H6
ARO:3001364	AJO16042.1	SHV-182 [*Klebsiella pneumoniae*]	A0A0C5C1Y7
ARO:3003156	AJO16047.1	SHV-189 [*Klebsiella pneumoniae*]	A0A0C5C1Z0
ARO:3001204	BAM28879.1	SHV-167 [*Klebsiella pneumoniae*]	I7GSH3
ARO:3002589	CAE50925.1	AAC(6′)-Iid [*Enterococcus hirae*]	Q70E72
ARO:3001337	CAQ03504.1	SHV-99 [*Klebsiella pneumoniae*]	B7FDD8
ARO:3003155	CEA29751.1	SHV-188 [*Klebsiella pneumoniae*]	A0A0A1ISX2

**Table 3:** J7I2U9 and J7I2V5 β-lactamase isoforms specific peptides. Peptides exclusively related to J7I2U9 and J7I2V5 β-lactamase isoforms are reported.

Protein.Entry	Protein Description	Uniprot Accession	Peptides Included Exclusively	RT	
				Mean	%CV
ARO:3001188	SHV-149; [*Klebsiella pneumoniae*]	J7I2U9	LSES**R**LSGSVGMIEMDLASGR	63.23	1.28
			LSGSVGMIEMDLASGR	72.80	1.14
			LSGSVGMIEMDLASGRTLTAWR	73.01	1.07
			SVLPAGWFIADKTGAGER	65.34	1.16
			TGAGERGAR	79.06	0.95
ARO:3001193	SHV-154; [*Klebsiella pneumoniae*]	J7I2V5	LSES**Q**LSGSVGMIEMDLASGR	64.68	1.15
			LSESQLSGSVGMIEMDLASGRTLTAWR	91.27	0.76

## References

[1] Cox G., Wright G.D. (2013). Intrinsic antibiotic resistance: Mechanisms, origins, challenges and solutions. Int. J. Med. Microbiol..

[2] Olivares J., Bernardini A., Egarcia-Leon G., Corona F., Sanchez M.B., Martínez J. (2013). The intrinsic resistome of bacterial pathogens. Front. Microbiol..

[3] Bhullar K., Waglechner N., Pawlowski A., Koteva K., Banks E.D., Johnston M.D., Balrton H.A., Wright G.D. (2012). Antibiotic resistance is prevalent in an isolated cave microbiome. PLoS ONE.

[4] Zhang X., Li L., Butcher J., Stintzi A., Figeys D. (2019). Advancing functional and translational microbiome research using meta-omics approaches. Microbiome.

[5] Soggiu A., Piras C., Mortera S.L., Alloggio I., Urbani A., Bonizzi L., Roncada P. (2016). Unravelling the effect of clostridia spores and lysozyme on microbiota dynamics in Grana Padano cheese: A metaproteomics approach. J. Proteom..

[6] Mortera S.L., Soggiu A., Vernocchi P., Del Chierico F., Piras C., Carsetti R., Marzano V., Britti D., Urbani A., Roncada P. (2019). Metaproteomic investigation to assess gut microbiota shaping in newborn mice: A combined taxonomic, functional and quantitative approach. J. Proteom..

[7] Soggiu A., Piras C., Gaiarsa S., Bendixen E., Panitz F., Bendixen C., Sassera D., Brasca M., Bonizzi L., Roncada P. (2016). Draft genome sequence of Clostridium tyrobutyricum strain DIVETGP, isolated from cow’s milk for Grana Padano production. Genome Announc..

[8] Hogan J.S., National Mastitis Council (1999). Laboratory Handbook on Bovine Mastitis.

[9] Brewster J.D., Paul M. (2016). Short communication: Improved method for centrifugal recovery of bacteria from raw milk applied to sensitive real-time quantitative PCR detection of *Salmonella* spp.. J. Dairy Sci..

[10] Wiśniewski J.R., Zougman A., Nagaraj N., Mann M. (2009). Universal sample preparation method for proteome analysis. Nat. Methods.

[11] Distler U., Kuharev J., Navarro P., Tenzer S. (2016). Label-free quantification in ion mobility–enhanced data-independent acquisition proteomics. Nat. Protoc..

[12] Marini F., Carregari V.C., Greco V., Ronci M., Iavarone F., Persichilli S., Castagnola M., Urbani A., Pieroni L. (2020). Exploring the HeLa Dark Mitochondrial Proteome. Front. Cell Dev. Biol..

[13] Jia B., Raphenya A.R., Alcock B., Waglechner N., Guo P., Tsang K.K., Lago B.A., Dave B.M., Pereira S., Sharma A.N. (2017). CARD 2017: Expansion and model-centric curation of the comprehensive antibiotic resistance database. Nucleic Acids Res..

[14] Alcock B.P., Raphenya A.R., Lau T.T.Y., Tsang K.K., Bouchard M., Edalatmand A., Huynh W., Nguyen A.-L.V., Cheng A.A., Liu S. (2020). CARD 2020: Antibiotic resistome surveillance with the comprehensive antibiotic resistance database. Nucleic Acids Res..

[15] Singh R.G., Tanca A., Palomba A., Van Der Jeugt F., Verschaffelt P., Uzzau S., Martens L., Dawyndt P., Mesuere B. (2019). Unipept 4.0: Functional Analysis of Metaproteome Data. J. Proteome Res..

[16] World Health Organization (2014). Antimicrobial Resistance Global Report on Surveillance.

[17] Noyes N.R., Yang X., Linke L.M., Magnuson R.J., Dettenwanger A., Cook S., Geornaras I., Woerner E.D., Gow S.P., McAllister A.T. (2016). Resistome diversity in cattle and the environment decreases during beef production. Elife.

[18] Liu J., Taft D.H., Maldonado-Gomez M.X., Johnson D., Treiber M.L., Lemay D.G., Depeters E.J., Mills D.A. (2019). The fecal resistome of dairy cattle is associated with diet during nursing. Nat. Commun..

[19] Noyes N.R., Yang X., Linke L.M., Magnuson R.J., Cook S.R., Zaheer R., Yang H., Woerner D.R., Geornaras I., McArt J.A. (2016). Characterization of the resistome in manure, soil and wastewater from dairy and beef production systems. Sci. Rep..

[20] Michael G.B., Freitag C., Wendlandt S., Eidam C., Feßler A.T., Lopes G.V., Kadlec K., Schwarz S. (2015). Emerging issues in antimicrobial resistance of bacteria from food-producing animals. Future Microbiol..

[21] Rovira P., McAllister T., Lakin S.M., Cook S.R., Doster E., Noyes N.R., Weinroth M.D., Yang X., Parker J.K., Boucher C. (2019). Characterization of the microbial resistome in conventional and “raised without antibiotics” beef and dairy production systems. Front. Microbiol..

[22] Cuny C., Arnold P., Hermes J., Eckmanns T., Mehraj J., Schoenfelder S., Ziebuhr W., Zhao Q., Wang Y., Feßler A.T. (2017). Occurrence of cfr-mediated multiresistance in staphylococci from veal calves and pigs, from humans at the corresponding farms, and from veterinarians and their family members. Vet. Microbiol..

[23] Tolle A. (1980). The Microflora of the Udder. In: Factors Influencing the Bacteriological Quality of Raw Milk. Int. Dairy J..

[24] Derakhshani H., Fehr K.B., Sepehri S., Francoz D., De Buck J., Barkema H.W., Plaizier J.C., Khafipour E. (2018). Invited review: Microbiota of the bovine udder: Contributing factors and potential implications for udder health and mastitis susceptibility. J. Dairy Sci..

[25] Hale O.J., Morris M., Jones B., Reynolds C.K., Cramer R. (2019). Liquid Atmospheric Pressure Matrix-Assisted Laser Desorption/Ionization Mass Spectrometry Adds Enhanced Functionalities to MALDI MS Profiling for Disease Diagnostics. ACS Omega.

[26] Piras C., Ceniti C., Hartmane E., Costanzo N., Morittu V.M., Roncada P., Britti D., Cramer R. (2020). Rapid liquid AP-MALDI MS profiling of lipids and proteins from goat and sheep milk for speciation and colostrum analysis. Proteomes.

[27] Oikonomou G., Machado V.S., Santisteban C., Schukken Y.H., Bicalho R.C. (2012). Microbial diversity of bovine mastitic milk as described by pyrosequencing of metagenomic 16s rDNA. PLoS ONE.

[28] Kuehn J.S., Gorden P.J., Munro D., Rong R., Dong Q., Plummer P.J., Wang C., Phillips G.J. (2013). Bacterial community profiling of milk samples as a means to understand culture-negative bovine clinical mastitis. PLoS ONE.

[29] Oikonomou G., Bicalho M.L., Meira E., Rossi R.E., Foditsch C., Machado V.S., Teixeira A.G.V., Santisteban C., Schukken Y.H., Bicalho R.C. (2014). Microbiota of cow’s milk; distinguishing healthy, sub-clinically and clinically diseased quarters. PLoS ONE.

[30] Nagaraj N., Kulak N.A., Cox J., Neuhauser N., Mayr K., Hoerning O., Vorm O., Mann M. (2012). System-wide perturbation analysis with nearly complete coverage of the yeast proteome by single-shot ultra HPLC runs on a bench top orbitrap. Mol. Cell. Proteom..

[31] Chavez M.V., Caicedo L.D., Castillo J.E. (2019). Occurrence of β-Lactamase-Producing Gram-Negative Bacterial Isolates in Water Sources in Cali City, Colombia. Int. J. Microbiol..

[32] Adelowo O.O., Ikhimiukor O.O., Knecht C., Vollmers J., Bhatia M., Kaster A.-K., Müller J.A. (2020). A survey of extended-spectrum beta-lactamase-producing Enterobacteriaceae in urban wetlands in southwestern Nigeria as a step towards generating prevalence maps of antimicrobial resistance. PLoS ONE.

[33] Adesoji A.T., Ogunjobi A.A. (2016). Detection of extended spectrum beta-lactamases resistance genes among bacteria isolated from selected drinking water distribution channels in southwestern Nigeria. BioMed Res. Int..

[34] Mesa R.J., Blanc V., Blanch A.R., Cortés P., Gonzalez J.J., Lavilla S., Miró E., Muniesa M., Saco M., Tórtola M.T. (2006). Extended-spectrum β-lactamase-producing Enterobacteriaceae in different environments (humans, food, animal farms and sewage). J. Antimicrob. Chemother..

[35] Tenover F.C., Filpula D., Phillips K.L., Plorde J.J. (1988). Cloning and sequencing of a gene encoding an aminoglycoside 6′-N-acetyltransferase from an R factor of Citrobacter diversus. J. Bacteriol..

